# Supplementary material for: The causal relationship between the human gut microbiota and pyogenic arthritis: a Mendelian randomization study
Source: Front Cell Infect Microbiol. 2024 Nov 26;14:1452480. doi: 10.3389/fcimb.2024.1452480 (PMC11629706; doi:10.3389/fcimb.2024.1452480)
Supplement: Supplementary file 1 [file DataSheet1.zip › mendelian test/ebi-a-GCST90027587.csv_leave_one_out.pdf]

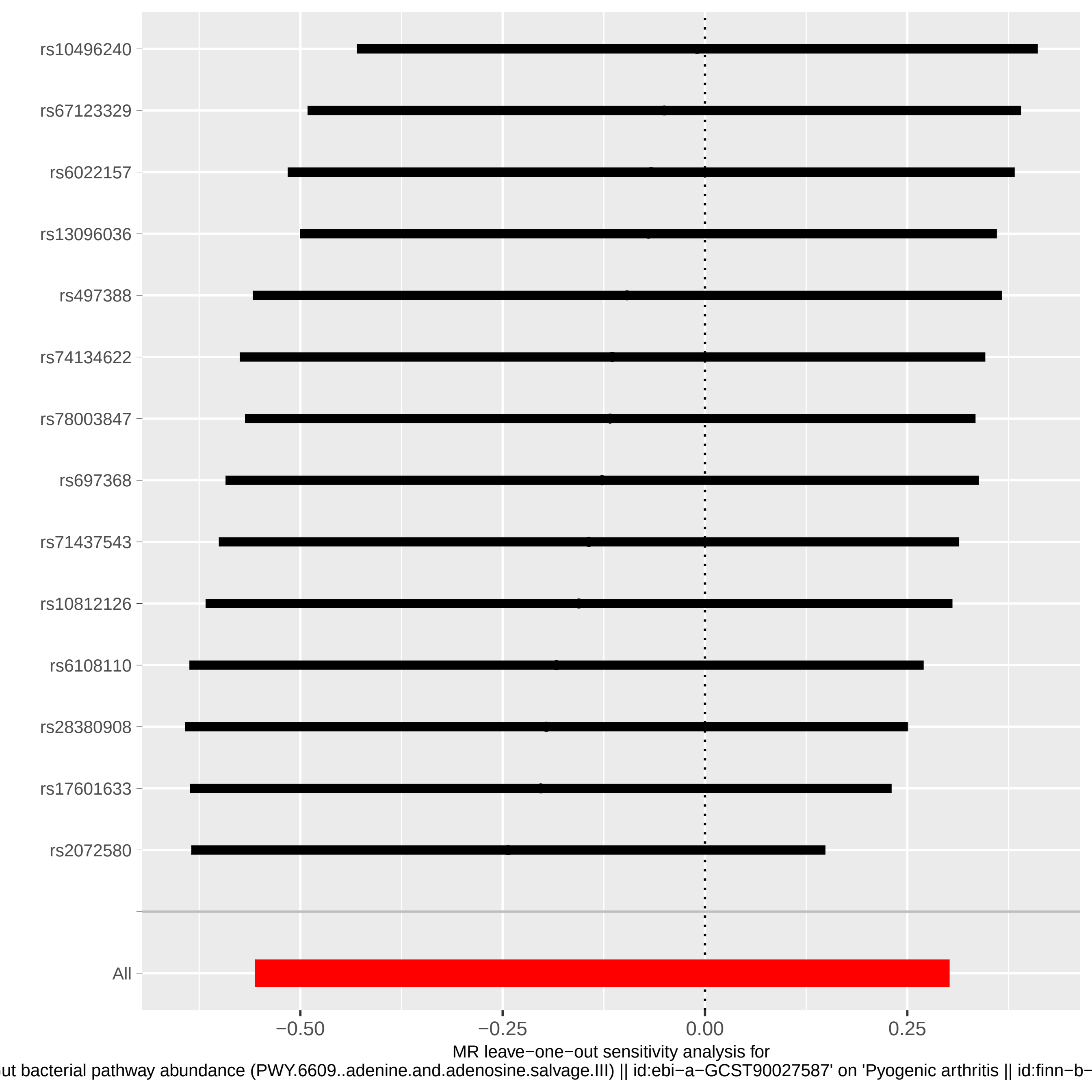

rs10496240
rs67123329
rs6022157
rs13096036
rs497388
rs74134622
rs78003847
rs697368
rs71437543
rs10812126
rs6108110
rs28380908
rs17601633
rs2072580
All
−0.50
−0.25
0.00
0.25
MR leave−one−out sensitivity analysis for
ut bacterial pathway abundance (PWY.6609..adenine.and.adenosine.salvage.III) || id:ebi−a−GCST90027587' on 'Pyogenic arthritis || id:finn−b−
